# Computational investigation of potential dosing schedules for a switch of medication from warfarin to rivaroxaban—an oral, direct Factor Xa inhibitor

**DOI:** 10.3389/fphys.2014.00417

**Published:** 2014-11-07

**Authors:** Rolf Burghaus, Katrin Coboeken, Thomas Gaub, Christoph Niederalt, Anke Sensse, Hans-Ulrich Siegmund, Wolfgang Weiss, Wolfgang Mueck, Takahiko Tanigawa, Jörg Lippert

**Affiliations:** ^1^Bayer HealthCareWuppertal, Germany; ^2^Bayer Technology Services GmbHLeverkusen, Germany

**Keywords:** coagulation, combination therapy, mathematical modeling, pharmacodynamics, rivaroxaban, simulation, warfarin

## Abstract

The long-lasting anticoagulant effect of vitamin K antagonists can be problematic in cases of adverse drug reactions or when patients are switched to another anticoagulant therapy. The objective of this study was to examine *in silico* the anticoagulant effect of rivaroxaban, an oral, direct Factor Xa inhibitor, combined with the residual effect of discontinued warfarin. Our simulations were based on the recommended anticoagulant dosing regimen for stroke prevention in patients with atrial fibrillation. The effects of the combination of discontinued warfarin plus rivaroxaban were simulated using an extended version of a previously validated blood coagulation computer model. A strong synergistic effect of the two distinct mechanisms of action was observed in the first 2–3 days after warfarin discontinuation; thereafter, the effect was close to additive. Nomograms for the introduction of rivaroxaban therapy after warfarin discontinuation were derived for Caucasian and Japanese patients using safety and efficacy criteria described previously, together with the coagulation model. The findings of our study provide a mechanistic pharmacologic rationale for dosing schedules during the therapy switch from warfarin to rivaroxaban and support the switching strategies as outlined in the Summary of Product Characteristics and Prescribing Information for rivaroxaban.

## Introduction

Many patients who require long-term vitamin K antagonist (VKA; e.g., warfarin) therapy experience difficulties in maintaining a therapeutic international normalized ratio (INR) (Ageno et al., 2012). Some patients may benefit from switching to one of the direct oral anticoagulants, such as rivaroxaban, which have been approved for the management of several thromboembolic disorders, including the prevention of stroke and systemic embolism in adult patients with non-valvular atrial fibrillation and at least one other risk factor for stroke. Rivaroxaban has been approved in this indication in Europe and the United States (at a dose of 20 mg once daily) (Bayer Pharma, 2014; Janssen Pharmaceuticals Inc., 2014) and in Japan (at a dose of 15 mg once daily) (Bayer Yakuhin Ltd., 2012; Hori et al., 2012).

VKAs are indirect anticoagulants that target multiple enzymes in the coagulation cascade. Specifically, they inhibit vitamin K-dependent clotting factors including Factors II, VII, IX, and X, and they also inhibit the carboxylation of the anticoagulant proteins C and S; therefore, VKAs have the potential to be procoagulants (Ansell et al., 2008). The use of VKAs is associated with the need for frequent monitoring and dose adjustment (Ansell et al., 2008) to ensure that the INR reaches and remains within the recommended therapeutic range (Ansell et al., 2008; Douketis et al., 2008; Kearon et al., 2012). The INR, based on the prothrombin time (PT) coagulation test, was developed to provide a standardized measure of the anticoagulant effect of VKAs and is the basis of guidelines for warfarin therapy (Ageno et al., 2012). Target INR values established for VKA therapies are specific to the distinct mechanisms of action of these agents and cannot be translated to anticoagulants with different mechanisms of action. For example, INR values measured in the therapeutic range of rivaroxaban are significantly lower than those required under warfarin therapy, indicating that INR measurements are not valid in this case (Kubitza et al., 2005a,b; Ansell et al., 2008; Douketis et al., 2008; Kearon et al., 2012).

Dose–response relationships of VKAs may be influenced by genetic factors, drug–drug interactions, and the consumption of alcohol and foods containing vitamin K (Ansell et al., 2008). For example, mutations in the vitamin K oxide reductase gene lead to enzymes with varying sensitivities to warfarin inhibition. Different ethnic populations vary in the frequency with which these mutations occur and, therefore, require different warfarin doses to maintain a therapeutic INR.

After discontinuation of warfarin, the concentrations of active vitamin K-dependent clotting factors and anticoagulant proteins C and S increase slowly toward normal levels. Because all of these factors are generated and cleared slowly, coagulation behavior is normalized only several days after warfarin discontinuation. Therefore, transitioning between warfarin and rivaroxaban therapies must be managed carefully and must account for the slow onset and long washout of warfarin effects to minimize the risk of bleeding and thrombotic events (Faaij et al., 2001, 2002; Reiffel, 2004; Patel et al., 2011).

Rivaroxaban is an oral, direct Factor Xa inhibitor with a wide therapeutic window (Perzborn et al., 2005; Roehrig et al., 2005) and predictable pharmacokinetic (PK) and pharmacodynamic (PD) properties. Because of its mechanism of action, rivaroxaban influences routine coagulation assays such as the PT/INR and activated partial thromboplastin time (aPTT). Phase I studies have shown a close correlation between PT and rivaroxaban plasma levels (Kubitza et al., 2005a,b).

In a previous study, we used an ordinary differential equation (ODE)-based computer model for blood coagulation to assess the optimal balance between efficacy and safety scenarios for rivaroxaban (Burghaus et al., 2011). This model was based on prior knowledge about coagulation factor concentrations after anticoagulant administration and data from *ex vivo* clotting tests; the model structurally resembles other recently published models of the effect of rivaroxaban on coagulation (Orfeo et al., 2010, 2011).

When modeling coagulation behavior during the transition phase from warfarin to rivaroxaban, the long PK and PD decay of warfarin, in conjunction with the effect of both warfarin and rivaroxaban on coagulation characteristics such as PT, need to be considered.

Currently, there is a lack of clinical data regarding PD changes during the transition from VKA therapy to direct oral anticoagulants, including rivaroxaban. Therefore, data derived from modeling approaches will provide useful information for clinicians planning to investigate the switch of medication experimentally.

The computational model used in this study was composed of the coagulation model as published by Burghaus et al. (2011), and the warfarin decay model describing the time course of coagulation factor concentrations as a result of warfarin treatment or its discontinuation. This model was used to investigate changes in the combined coagulation effect during the switch from warfarin to rivaroxaban. Our objectives were to improve mechanistic understanding of the interaction between warfarin and rivaroxaban during the transition and to provide a mechanistic pharmacologic rationale for dosing schedules during the therapy switch, thereby supporting the switching strategies as outlined in the Summary of Product Characteristics and Prescribing Information for rivaroxaban (Bayer Pharma, 2014; Janssen Pharmaceuticals Inc., 2014).

## Materials and methods

### Model setup

**Figure 1:**
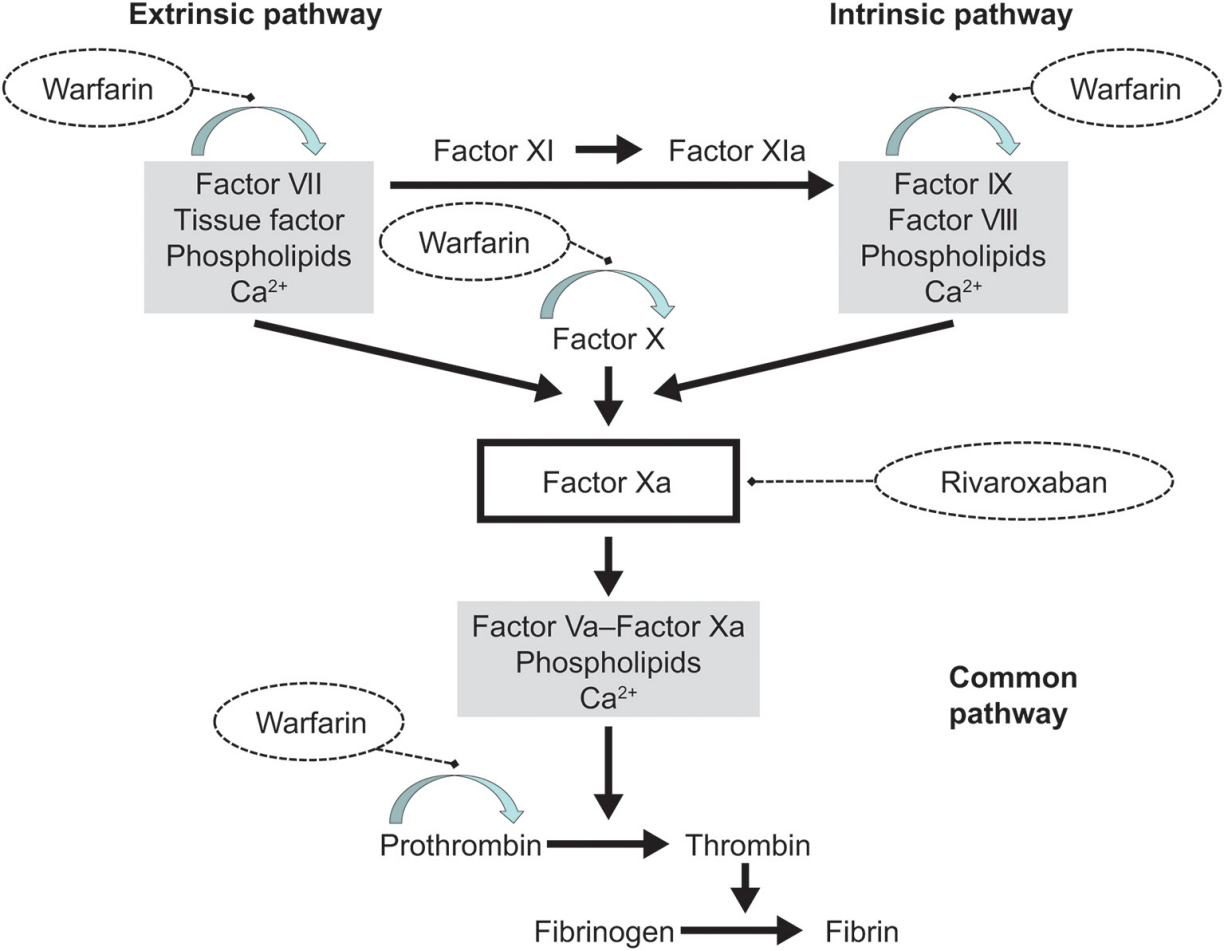
**Overview of the processes accounted for by the coagulation model**. The blood coagulation model represents the biochemical reactions that result in factor activation. The extrinsic and intrinsic pathways of the model lead to thrombin and fibrin formation downstream of Factor Xa, known as the common pathway. Drug action is represented by the competitive inhibition of Factor Xa by rivaroxaban and by down-regulation of the vitamin K-dependent synthesis (arched arrow) of the factors VII, IX, and X, and prothrombin (Factor II) by warfarin. These structural elements of the coagulation cascade, as well as proteins C and S (not depicted), formed the basis of the model by Burghaus et al. (2011).

We simulated the effect of warfarin monotherapy and the combined effects of warfarin and rivaroxaban using an ODE-based blood coagulation model that represents coagulation in *ex vivo* clotting tests with human plasma such as PT and aPTT; the model has been described previously (Burghaus et al., 2011) and is based largely on several published models (Kogan et al., 2001; Hockin et al., 2002; Anand et al., 2003; Bungay et al., 2003; Orfeo and Mann, 2005). The model, as described previously by Burghaus et al. (2011), takes into account both the intrinsic and extrinsic pathways of the coagulation cascade, as well as the common pathways leading to fibrin generation via thrombin (Figure 1). As such, it possesses some unique features that were not included in earlier models, such as a portfolio of drug action mechanisms. Study drugs were modeled by closely representing their anticoagulant properties (Burghaus et al., 2011). Supplementary Material 1—Model Pack, provides the complete model as implemented in MoBi® and all (Matlab®) codes used for simulations and generation of figures. This provides full insight into the processes of computational warfarin titration and the parameters used for the simulations.

By use of parameter studies, the model can be used to investigate scenarios of blood coagulation initiated by very weak triggers that cannot be investigated in a controlled manner in experimental assays. For the simulations in this study we used the previously described model and added a turnover model for the synthesis and decay of vitamin K-dependent Factors II, VII, IX, and X, and proteins C and S. Steady-state concentrations of these factors were set as previously reported in Burghaus et al. (2011). In the current study, model parameters that correspond to blood flow were set to zero.

The mechanism of action of rivaroxaban was already implemented in the previously described model (Burghaus et al., 2011). However, warfarin action had to be newly implemented: warfarin PK was not explicitly modeled. Both the steady-state effect of warfarin and its decay after discontinuation of therapy are represented by effective inhibition of synthesis rates of vitamin K-dependent coagulation factors in a standard turnover model. Parameters of the turnover model were chosen such that steady-state conditions (starting concentrations) for vitamin K-dependent factors of the turnover model corresponded to previously published data (Wittkowsky, 2003). No PK interactions between warfarin and rivaroxaban have been identified and, therefore, such potential interactions were not accounted for in the model (Food and Drug Administration, 2011; Johnson and Johnson Pharmaceutical Research and Development, 2011).

#### Turnover model for vitamin K-dependent coagulation factors

The turnover model for vitamin K-dependent coagulation factors (Table 1) describes the formation and clearance of the relevant proteins. Coagulation factor clearance is implemented as an exponential decay to reflect physiological conditions. The half-lives used in the model were as follows (Table 1C): Factor II, 57 h; Factor VII, 5 h; Factor IX, 25.5 h; Factor X, 37.5 h; protein C, 9 h; protein S, 60 h (Wittkowsky, 2003).

**Table 1:** **The warfarin decay model: (A) reaction; (B) species; (C) species half-lives**.

**(A) REACTIONS IN THE WARFARIN DECAY MODEL**		
**Reaction name**	**Stoichiometry**	**Kinetics**
RfII–decay	fII → inactive species^*^	fII × ln(2)/*t*_½_ fII
RfII–formation	Profactor^*^ → fII	(kfII × ln(2)/*t*_½_ fII)/(formation rate + 1)
RfIX–decay	fIX → inactive species^*^	fIX × ln(2)/*t*_½_ fIX
RfIX–formation	Profactor^*^ → fIX	(kfIX × ln(2)/*t*_½_ fIX)/(formation rate + 1)
RfVII–decay	fVII → inactive species^*^	fVII × ln(2)/*t*_½_ fVII
RfVII–formation	Profactor^*^ → fVII	(kfVII × ln(2)/*t*_½_ fVII)/(formation rate + 1)
R-warfarin–decay	R-warfarin → inactive species^*^	R-warfarin × ln(2)/*t*_½_ R-warfarin
S-warfarin–decay	S-warfarin → inactive species^*^	S-warfarin × ln(2)/*t*_½_ S-warfarin
RfX–decay	fX → inactive species^*^	fX × ln(2)/*t*_½_ fX
RfX–formation	Profactor^*^ → fX	(kfX × ln(2)/*t*_½_ fX)/(formation rate + 1)
Protein C–decay	Protein C → inactive species^*^	Protein C × ln(2)/*t*_½_ Protein C
Protein C–formation	Profactor^*^ → Protein C	(kProtein C × ln(2)/*t*_½_ Protein C)/(formation rate + 1)
Protein S–decay	Protein S → inactive species^*^	Protein S × ln(2)/*t*_½_ Protein S
Protein S–formation	Profactor^*^ → Protein S	(kProtein S × ln(2)/*t*_½_ Protein S)/(formation rate + 1)
**(B) KEY PARTICIPANTS IN THE WARFARIN DECAY MODEL**		
**Species**	**Initial concentration (M)**	
fII	1.40 × 10^−6^	
fIX	9.00 × 10^−8^	
fVII	1.00 × 10^−8^	
Effective R-warfarin/ S-warfarin^*^	Fitted to obtain start INR	
fX	1.60 × 10^−7^	
Protein C	6.00 × 10^−8^	
Protein S	1.40 × 10^−7^	
Protein C/S binding sites on phospholipids	3.60 × 10^−6^	
**(C) TYPICAL HALF-LIFE (*t*_½_) FOR SPECIES IN THE WARFARIN DECAY MODEL**		
**Species (half-life)**	**Time (s)**	
*t*_½_ fII	205,200 (57 h)	
*t*_½_ fIX	91,800 (25.5 h)	
*t*_½_ fVII	18,000 (5 h)	
*t*_½_ fX	135,000 (37.5 h)	
*t*_½_ protein C	32,400 (9 h)	
*t*_½_ protein S	216,000 (60 h)	
*t*_½_ R-warfarin	162,000	
*t*_½_ S-warfarin	104,400^†^	

*Note for Table A: For the calculation of the formation rate, see section on Turnover model for vitamin K-dependent coagulation factors in the Materials and Methods*.

* *Indicates a ‘boundary species’ that has its value kept constant during the simulation. f, Factor; k, kinetic rate constant; R, reaction; t_½_, half-life*.

Note for Table B:

* *Effective R-warfarin and S-warfarin concentrations are in arbitrary units reflecting the fraction of remaining warfarin*.

*Factor concentrations (II, VII, IX, X) are average plasma concentrations taken from Hockin et al. (2002). Protein C and S concentrations were taken from Bungay et al. (2003); protein S was corrected to reflect the free plasma concentration (40% of total) reported in the protein C monograph available online at www.chromogenix.com. f, Factor; INR, international normalized ratio*.

Note for Table C:

† *74,897 for Japanese patients. f, Factor*.

Therapeutic warfarin is considered to be applied in a racemic 1:1 mixture of R-warfarin and S-warfarin. The effect of both warfarin components was implemented as a pre-factor W reducing the formation rates of coagulation factors according to the following equation, with R-warfarin and S-warfarin representing the respective effective concentrations (in arbitrary units; see below and Supplementary Material 1):

$$\text{W}={\left({\left[\text{R-warfarin}+\text{3}.\text{25}\times \text{S-warfarin}\right]}^{2}+1\right)}^{-1}$$

**Figure 2:**
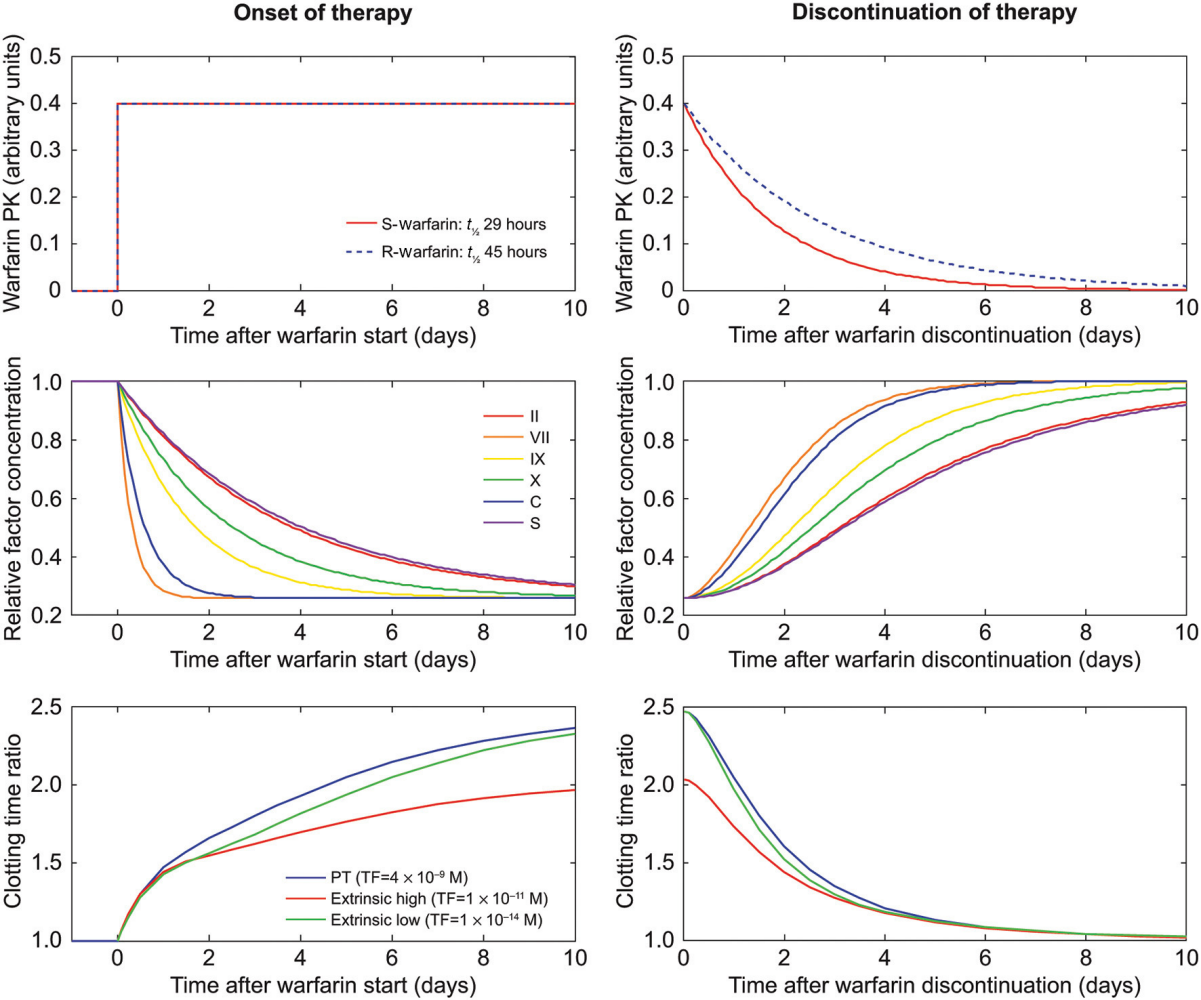
**Dynamic behavior of the warfarin decay model**. Graphs on the left side show the sudden onset of warfarin therapy simulated for Caucasians. Graphs on the right side show warfarin discontinuation, eventually leading to normal values for the coagulation system. Upper graphs show the values of R-warfarin and S-warfarin (arbitrary units); middle graphs show the corresponding evolution of factor concentrations; and lower graphs show coagulation time (relative to an untreated scenario) for different triggers (Table 2) in the coagulation model, depending on the factor concentrations from the middle graphs. PK, pharmacokinetics; PT, prothrombin time; TF, tissue factor; *t*_½_, half-life.

This equation takes into account that S-warfarin is, on average, 3.25-fold more potent than R-warfarin, with the square having its rationale in the two steps inhibited by warfarin in the hydroquinone/epoxide cycle of the vitamin K process (Wittkowsky, 2003). S-warfarin and R-warfarin decay with half-lives of 29 and 45 h, respectively (Wittkowsky, 2003). Discontinuation of warfarin is modeled by simulating the decay of R-warfarin and S-warfarin, resulting in an increase in vitamin K-dependent factor production rates and thus factor concentrations. Figure 2 shows the obtained warfarin decay and factor increase plots over a 10-day period (240 h).

The parameters R-warfarin and S-warfarin of the warfarin decay model (Table 1), which are considered to be expressed in arbitrary units of unknown proportionality to *in vivo* concentrations, were adjusted to reach the INR (corresponding to steady-state warfarin treatment) that was chosen as a starting point for the respective warfarin discontinuation simulation. For example, the warfarin parameter resulting in an INR of 2.5 and relative factor concentrations of approximately 30% was found to be 0.4 (Figure 2), in line with actual clinical observations (Ferreira et al., 2002).

The warfarin action and decay model was programmed in MoBi® 2.3 (Bayer Technology Services, Leverkusen, Germany) (Bayer Technology Services, 2010), as were the already published model components (see listing of model parameters in Table 1).

### Simulations

#### Simulation of coagulation status during switching

In general, to simulate the coagulation status during a switch from warfarin to rivaroxaban, we focused on drug–drug combination effects as characterized by the PT test and trigger scenarios considered relevant for bleeding risks as well as antithrombotic efficacy (Table 2). The latter scenarios have been established and tested previously (Burghaus et al., 2011). In clinical practice PT values are commonly reported as INR values to indicate the anticoagulant effect of VKAs. Therefore, for comparison with clinical data, simulated PT test results were translated into INR values assuming an ISI (International Sensitivity Index; a characteristic of *in vitro* PT reagents used for calibration of experimental data) value for our *in silico* PT assay of 1.0.

**Table 2:** **Coagulation scenarios used for simulations**.

**Scenario**	**Tissue factor concentration (M)**
Prothrombin time test (extrinsic)	4.0 × 10^−9^
Extrinsic strong	10^−11^
Extrinsic weak	10^−14^

Starting conditions for simulations of switching were set by adjusting the effective concentration of R- and S-warfarin (and the resulting steady-state concentrations of vitamin K-dependent factors) to represent steady-state warfarin therapies at a chosen INR value, including INRs below (1.5), within (2.5), and above (3.5) the therapeutic window. For a given time point after discontinuation of warfarin therapy, the effective concentrations of R- and S-warfarin were calculated using the decay model.

To evaluate the model, we first simulated the decay of warfarin without addition of rivaroxaban and compared it with data from a clinical study of warfarin discontinuation. We then investigated the interaction with rivaroxaban as characterized by a PT test (and the resulting INR values) by re-running the warfarin decay simulations with added rivaroxaban, testing a broad range of rivaroxaban concentrations.

#### Simulations of the therapeutic corridor in Caucasian patients

To evaluate the safety and efficacy of rivaroxaban combined with residual warfarin action in virtual Caucasian patients, we used simulation to identify threshold rivaroxaban concentrations leading to the same clotting times in previously established trigger scenarios (Burghaus et al., 2011), as obtained for warfarin monotherapy at INR values of 1.5, 2.0, 4.0, and 5.0. These INR values are within the INR range considered to be of clinical relevance (Ageno et al., 2012) for the following reasons: the target therapeutic range of INRs for anticoagulation is generally between 2.0 and 3.0; special patient populations may need to be adjusted to lower INR ranges; and an INR higher than 3.0 is not uncommon in patients receiving VKA therapy.

The strong extrinsic trigger (TF = 10^−11^ M) was assumed to be the relevant safety scenario. The weak extrinsic trigger (TF = 10^−14^ M) was considered to be the relevant efficacy scenario. A safe rivaroxaban exposure was assumed to provide faster clotting after the strong trigger than warfarin therapies with INR values of 4.0 and 5.0 for patients with a high and a normal risk of bleeding, respectively. An efficacious rivaroxaban scenario was assumed to extend clotting times after the weak trigger beyond those times obtained for warfarin therapies at INR values of 1.5 and 2.0 for patients with normal and high thrombotic risk, respectively. These safety and efficacy thresholds were calculated for each time point during the first 7 days after warfarin discontinuation. The impact of INR at the time of discontinuation was evaluated by simulating initial INR values of 1.5, 2.5, and 3.5. The impact of warfarin PK variability was studied by simulating different half-lives. We used typical slow, median, and fast half-lives for S- and R-warfarin (52, 29, and 18 h for S-warfarin, and 70, 45, and 20 h for R-warfarin, respectively).

#### Simulations of the therapeutic corridor in Japanese patients

We also performed simulations in virtual Japanese patients. In the Japanese population, the variability of clearance via cytochrome P450 2C9 is lower than in most other populations (Takahashi et al., 2006). When simulating warfarin discontinuation for the Japanese population, we adjusted the half-life of S-warfarin by multiplying the typical values used for Caucasians with a relative clearance (469.4/654.3) for Japanese versus Caucasian patients as determined previously (Takahashi et al., 2006). The only well-documented difference in the coagulation cascade in the Japanese population is a reduced level of Factor VII pre-activation of 0.5% as compared with 1.0% in Caucasians (Kario et al., 1994); this difference was accounted for in the Japanese model. In the simulations of Japanese patients, R-warfarin and coagulation factor half-lives, as well as their variability ranges, were the same as for the Caucasian simulations.

#### Impact of differences in coagulation factor expression status

To assess the robustness of our study approach for differences in coagulation factor expression status (e.g., as a consequence of genetic predisposition), we repeated the above simulation program for a broad panel of *in silico* patients with different factor abnormalities (over- or under-expression in Factors I, V, VIII, and XI; Table 3 and Supplementary Material 2). These factors were chosen because they are independent of warfarin. Consequently, their levels do not vary depending on the dose of warfarin and they can be used to evaluate the sensitivity of the model.

**Table 3:** **Simulation program for patients with factor abnormalities**.

	**Factor I**	**Factor V**	**Factor VIII**	**Factor XI**
**Normal patient**	1	1	1	1
**Factor I deficient**	0.15	1	1	1
	0.5	1	1	1
	0.75	1	1	1
	2	1	1	1
	5	1	1	1
**Factor V deficient**	1	0.01	1	1
	1	0.1	1	1
	1	0.5	1	1
	1	0.75	1	1
	1	2	1	1
	1	5	1	1
**Factor VIII deficient**	1	1	0.01	1
	1	1	0.1	1
	1	1	0.5	1
	1	1	0.75	1
	1	1	2	1
	1	1	5	1
**Factor XI deficient**	1	1	1	0.01
	1	1	1	0.1
	1	1	1	0.5
	1	1	1	0.75
	1	1	1	2
	1	1	1	5

*The values presented are ratios of normal values*.

## Results

**Figure 3:**
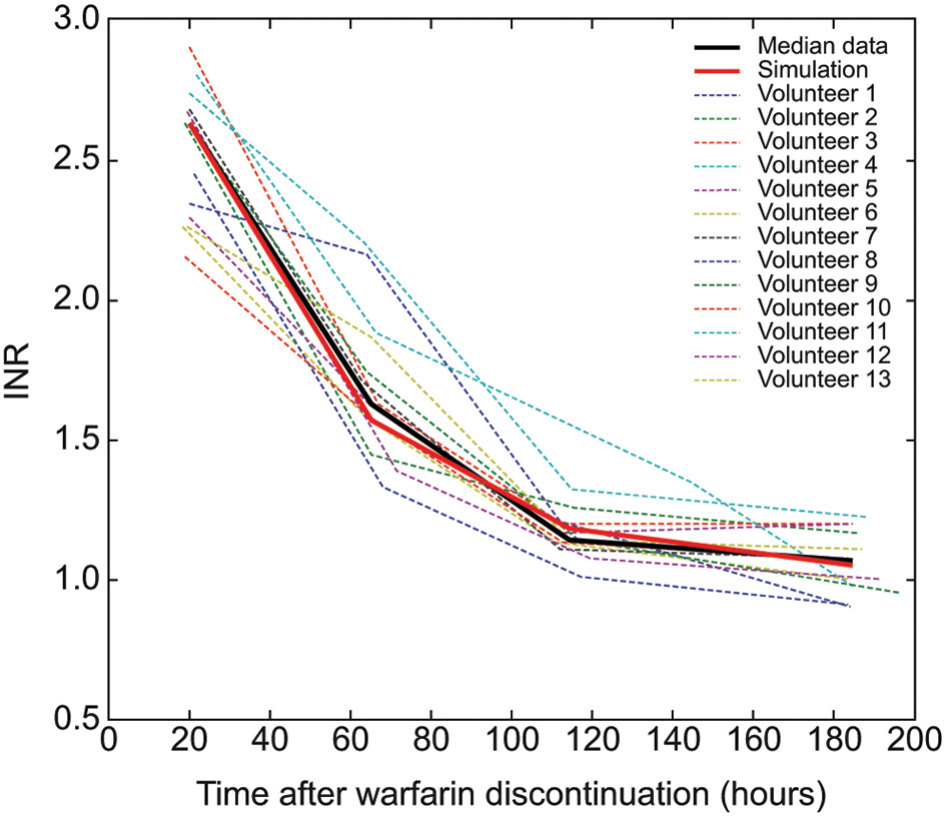
**Validation of the warfarin decay model**. Thin colored lines represent the international normalized ratio (INR) data of 13 individual patients after discontinuation of warfarin therapy (White et al., 1995) (individual data courtesy of R.H. White, UC Davis, California, USA); the thick black line provides the associated median at every time point; the thick red line describes the simulated INR during warfarin decay.

To obtain initial conditions for the simulation of the discontinuation of warfarin therapy the effect of adding warfarin and its effect on factor synthesis was simulated until steady-state conditions were reached. Figure 2 illustrates the effect of setting the effective warfarin concentration to 0.4 (upper left panel) on the temporal change of relative factor concentrations (middle left panel; for all three trigger scenarios) and the resulting increase in clotting times (lower left panel) over 10 days. The simulation of the discontinuation of warfarin treatment (Figure 2) showed a slow exponential decay of the effective S- and R-warfarin concentrations (Figure 2, upper right panel). Concentrations of vitamin K-dependent factors returned slowly to baseline values, defined as values in the absence of warfarin treatment (1.0; Figure 2, middle right panel), although some factors took more than 10 days to reach 100% of baseline values. Similar timescales were obtained for the decay of clotting time prolongation (Figure 2, lower right panel). Simulation data of coagulation factors were a good fit to clinical data from a warfarin discontinuation study (White et al., 1995), as shown in Figure 3.

**Figure 4:**
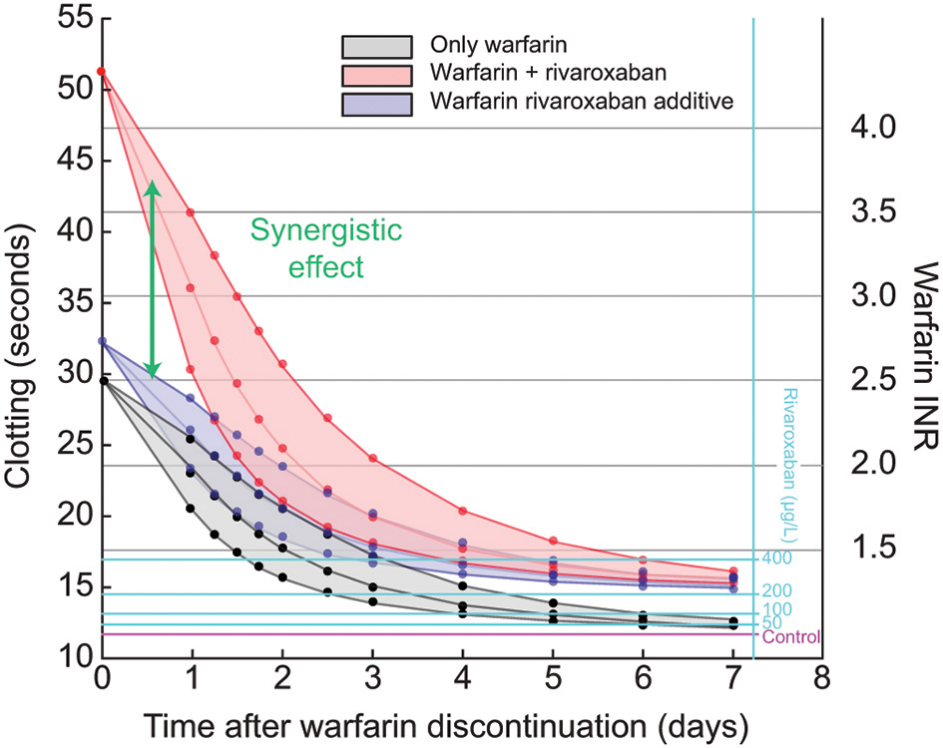
**Synergistic effect of warfarin and rivaroxaban (200 μg/L) after warfarin discontinuation**. Black lines show the prothrombin time (PT) as a function of time after warfarin discontinuation, starting at an international normalized ratio (INR) of 2.5, simulated for Caucasians. Rivaroxaban exposure of 200 μg/L causes a PT prolongation of about 3 s in a patient not on warfarin therapy. Blue lines show the hypothetical additive effect of warfarin and rivaroxaban exposure of 200 μg/L. Red lines show the actual simulation of the effect of decaying warfarin plus rivaroxaban (synergistic effect). The center lines are simulated for typical warfarin half-life; lower and upper lines show the same experiments for fast and slow warfarin decay, respectively, using half-lives as reported previously (Wittkowsky, 2003). Light blue lines show the effect of different degrees of rivaroxaban exposure for comparison.

Next, we investigated the impact of different warfarin half-lives on the decay of INR (Figure 4). We simulated typical slow, median, and fast half-lives for S- and R warfarin (52, 29, and 18 h for S-warfarin, and 70, 45, and 20 h for R-warfarin, respectively) (see Methods section) and plotted time-dependent changes in INR over 7 days (Figure 4; black lines and gray shaded area). We simulated the effect of rivaroxaban on PT and INR by adding constant concentrations of rivaroxaban to the simulated decay of warfarin action. At the time of discontinuation, a concentration of 200 μg/L rivaroxaban increased PT values from approximately 30 s owing to the warfarin effect from an INR of 2.5 to more than 50 s (Figure 4; red lines and shaded area), indicating a synergistic effect. Rivaroxaban alone at a concentration of 200 μg/L without any warfarin effect prolonged PT by 3 s from 12 s to approximately 15 s (Figure 4; light blue horizontal lines). A putative, only additive interaction between warfarin and rivaroxaban would, therefore, only explain an increase of PT from 30 to 33 s (Figure 4; blue lines and shaded area).

**Figure 5:**
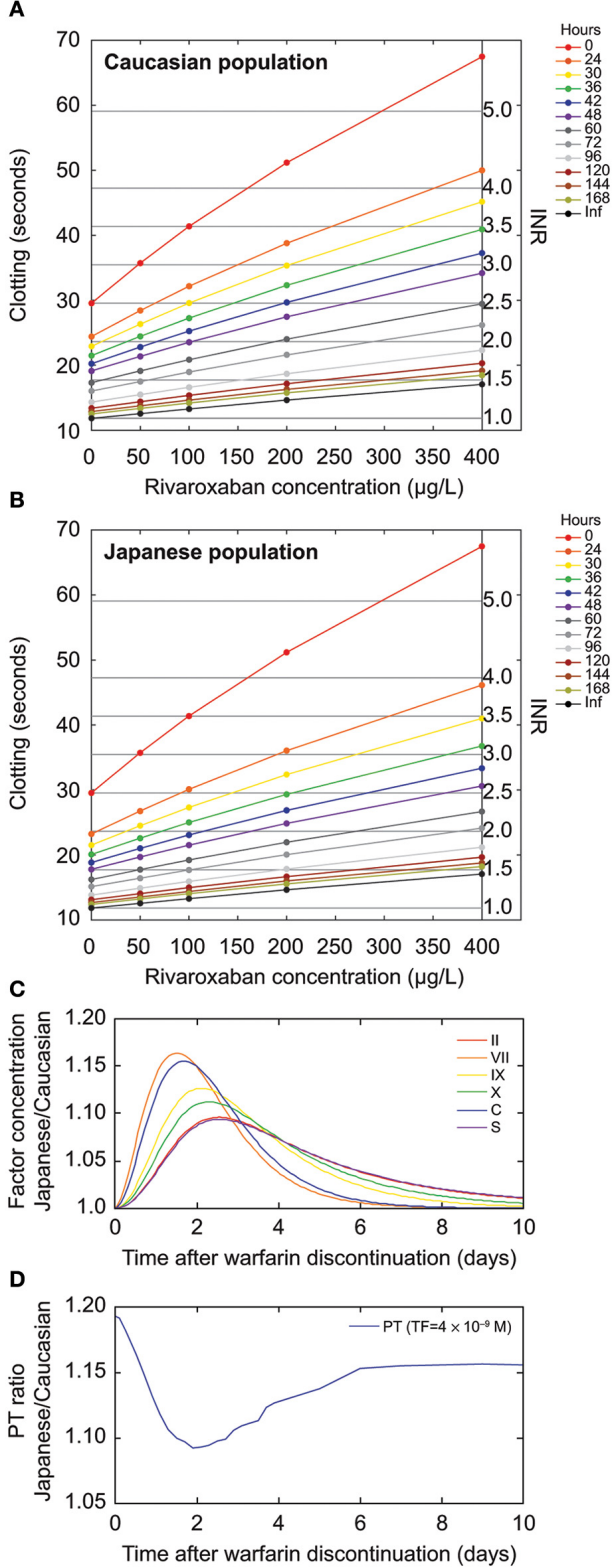
**Prothrombin time (PT) characteristics for rivaroxaban after warfarin discontinuation**. **(A,B)** Solid black line represents the effect of rivaroxaban without warfarin. The impact of rivaroxaban is stronger when rivaroxaban therapy is started early after warfarin discontinuation. **(A)** Modeled for the Caucasian population. **(B)** Modeled for the Japanese population. **(C,D)** Time course of **(C)** vitamin K-dependent factor concentration ratios and **(D)** PT ratio of simulated Japanese versus Caucasian patients. Vitamin K-dependent factors start from 40% of the normal for both Japanese and Caucasians. **(D)** The tissue factor (TF) value used to simulate PT was set at 4 × 10^−9^ M. The simulated Japanese normal PT is slightly higher than the simulated Caucasian normal PT because of reduced Factor VII preactivation, resulting in a ratio >1 after the warfarin effect has subsided (8–10 days). Inf, infinite time (after warfarin discontinuation); INR, international normalized ratio.

The slope of the PT versus rivaroxaban concentration relationship is a measure of rivaroxaban potency. A systematic analysis of the time dependency of this slope after warfarin discontinuation showed that the strongest synergism occurred directly after discontinuation (Figure 5A). For up to 72 h after discontinuation, the slope was clearly steeper than for rivaroxaban alone (*t* = inf; Figure 5A). A similar pattern was demonstrated for simulations with Japanese *in silico* patients (see Materials and Methods; Figure 5B), which also showed the faster warfarin decay in these patients as a consequence of the shorter S-warfarin half-life compared with Caucasian patients (see Materials and Methods). Differences between the Japanese and Caucasian populations were observed both at the level of the relative concentrations of individual coagulation factors (Figure 5C) as well as in the resulting relative PT values (Figure 5D). These analyses showed the biggest difference between Caucasian and Japanese patients at around 2 days after warfarin discontinuation.

**Figure 6:**
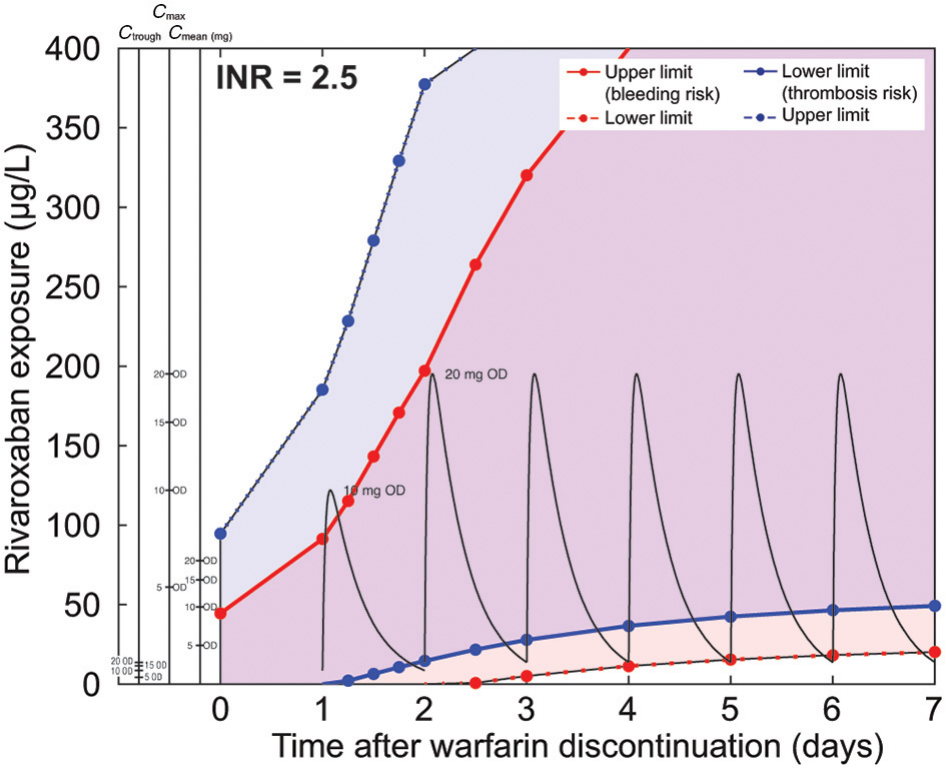
**Nomogram for a typical warfarin decay scenario starting at an international normalized ratio (INR) of 2.5 for the Caucasian *in silico* patient**. The x-axis describes the time after discontinuation of warfarin and the y-axis corresponds to rivaroxaban exposure. Blue and red lines correspond to the edges of the safety and efficacy regions. The strong extrinsic trigger (TF = 10^−11^ M) was assumed to be the relevant safety scenario. The weak extrinsic trigger (TF = 10^−14^ M) was considered to be the relevant efficacy scenario. Rivaroxaban exposure above the dashed red line is considered to be safe and exposure below the dashed blue line is considered to be efficacious for most patients. The region between the solid red and solid blue lines describes the exposure range to which patients with a high risk of bleeding or thrombosis should be dosed. Patients with a high risk of bleeding should stay below the exposure limit represented by the solid red line. Patients with a high risk of thrombosis should stay above the exposure limit represented by the solid blue line. Alternative y-axes convert rivaroxaban exposure into mean plasma concentration (*C*_mean_), maximum plasma concentration (*C*_max_), and minimum plasma concentration (*C*_trough_) values for the different rivaroxaban dosing regimens studied (5, 10, 15, and 20 mg once daily [OD]). Pharmacokinetic curves for rivaroxaban 10 and 20 mg OD are drawn in black. These nomograms do not account for pharmacokinetic variability or uncertainty in parameters. However, the potential impact of pharmacokinetic variability of rivaroxaban can also be evaluated from this graph although this is not depicted explicitly.

Next, we simulated a typical warfarin decay scenario over a 7-day period (168 h) for an initial INR of 2.5 at discontinuation for Caucasian patients and calculated efficacy and safety corridors for rivaroxaban exposure. These corridors were defined by clotting time thresholds for extrinsic triggers (Table 2) as obtained for treatment with warfarin only corresponding to INR values of 1.5, 2.0, 4.0, and 5.0. The results were translated into a nomogram (Figure 6) depicting the rivaroxaban concentration range compliant with the calculated thresholds. The nomogram illustrates that the therapeutic window for rivaroxaban is narrower at the time of discontinuation of warfarin and widens in the subsequent days before it reaches the full width as given for monotherapy without warfarin co-medication. To relate the exposure window to rivaroxaban PK, the y-axis was enriched with information about trough, mean, and peak concentrations for 5, 10, 15, and 20 mg once-daily doses of rivaroxaban. We plotted the mean PK profile resulting from a rivaroxaban dosing schedule starting with 10 mg once daily on day 1 after discontinuation, and continuing with rivaroxaban 20 mg once daily (the dose approved in Europe and the United States for the prevention of stroke and systemic embolism in patients with atrial fibrillation) (Figure 6). The superimposed PK profile shows that day-end levels were within the safety limit. Therefore, the graph demonstrates that this schedule is compliant with the *in silico* safety and efficacy criteria (red and blue lines, respectively).

**Figure 7:**
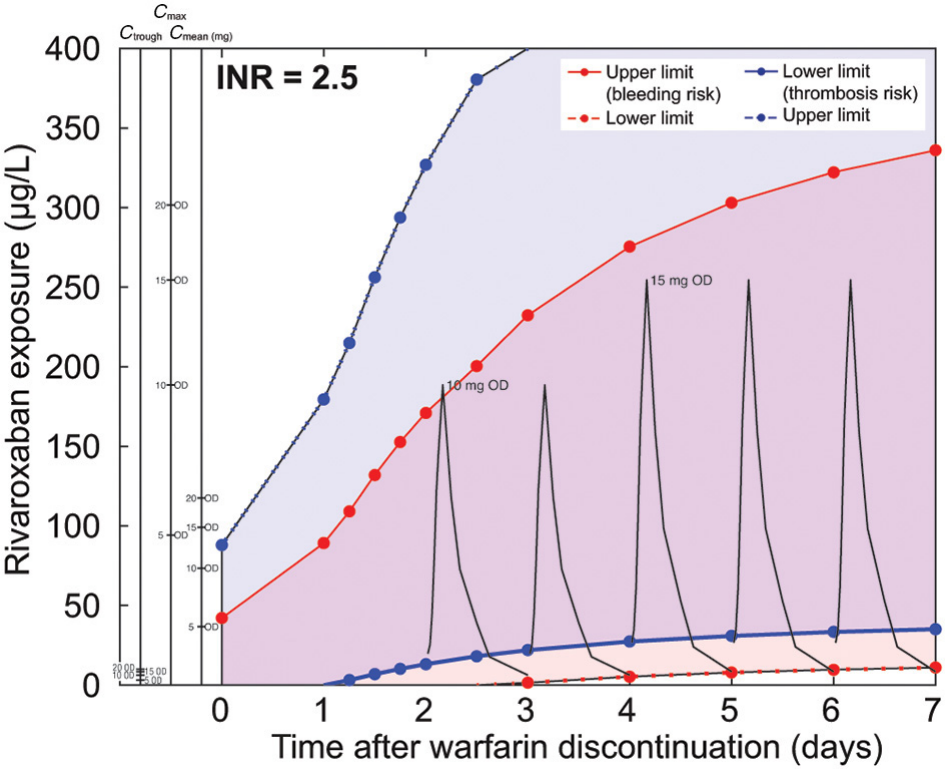
**Nomogram for a typical warfarin decay scenario starting at an international normalized ratio (INR) of 2.5 for the Japanese *in silico* patient**. Pharmacokinetic curves for rivaroxaban 10 and 15 mg OD are drawn in black. (Note: please refer to legend of Figure 6 for further details.)

The same type of simulation was performed for the safety and efficacy corridor for *in silico* Japanese patients starting at an INR of 2.5 (Figure 7). The mean rivaroxaban concentrations for two consecutive days with 10 mg once daily starting on day 2 after warfarin discontinuation followed by rivaroxaban 15 mg once daily doses comply with the calculated corridor. The information about mean, peak, and trough concentrations of rivaroxaban are also provided in the nomogram allowing assessment of safety and efficacy of other dosing schedules. For instance, a schedule starting on day 1 with 5 mg once daily, continuing with a daily increase to 10 mg once daily and finally 15 mg once daily on day 3, would also match the corridor.

**Figure 8:**
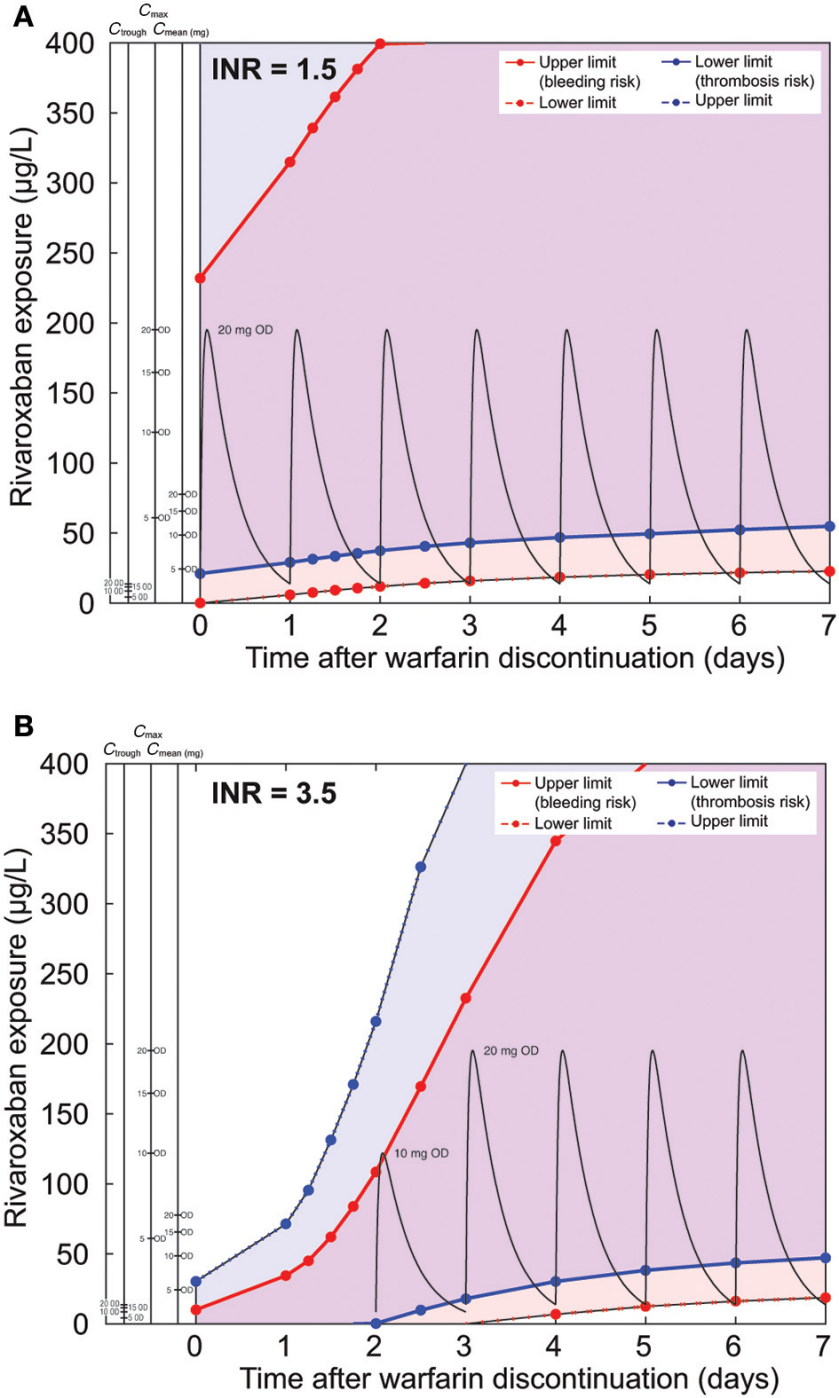
**Comparison of nomograms for patients with INR of (A) 1.5 and (B) 3.5 (typical warfarin decay is assumed)**. Pharmacokinetic curves for rivaroxaban 10 and 20 mg OD are drawn in black. For panel **(A)**, the dashed blue line representing the upper exposure limit for normal patients extends past the axis limits of the figure and is, therefore, not presented on the graph. (Note: please refer to legend of Figure 6 for further details.)

We also investigated the effect of different baseline INR levels at discontinuation. With an INR of 1.5 at discontinuation, we found that a flat rivaroxaban schedule of 20 mg once daily started already on day 0, when the next warfarin dose would be due, matched the *in silico* criteria (Figure 8A). For comparison we have plotted the same dosing schedule as for the simulation starting at an INR of 2.5. By design, the lower efficacy threshold was above 0 immediately after discontinuation leading to the need to dose rivaroxaban in order to stay within the corridor. With an INR of 3.5 at discontinuation, the safety and efficacy corridor was very narrow just after discontinuation (Figure 8B). As for simulations with an initial INR of 2.5, the superimposed PK profile shows that day-end levels were within the safety limit. The nomogram suggests starting with rivaroxaban 10 mg once daily only on day 2 after warfarin discontinuation when the therapeutic window has already broadened, followed by rivaroxaban 20 mg once daily from day 3 onwards.

**Figure 9:**
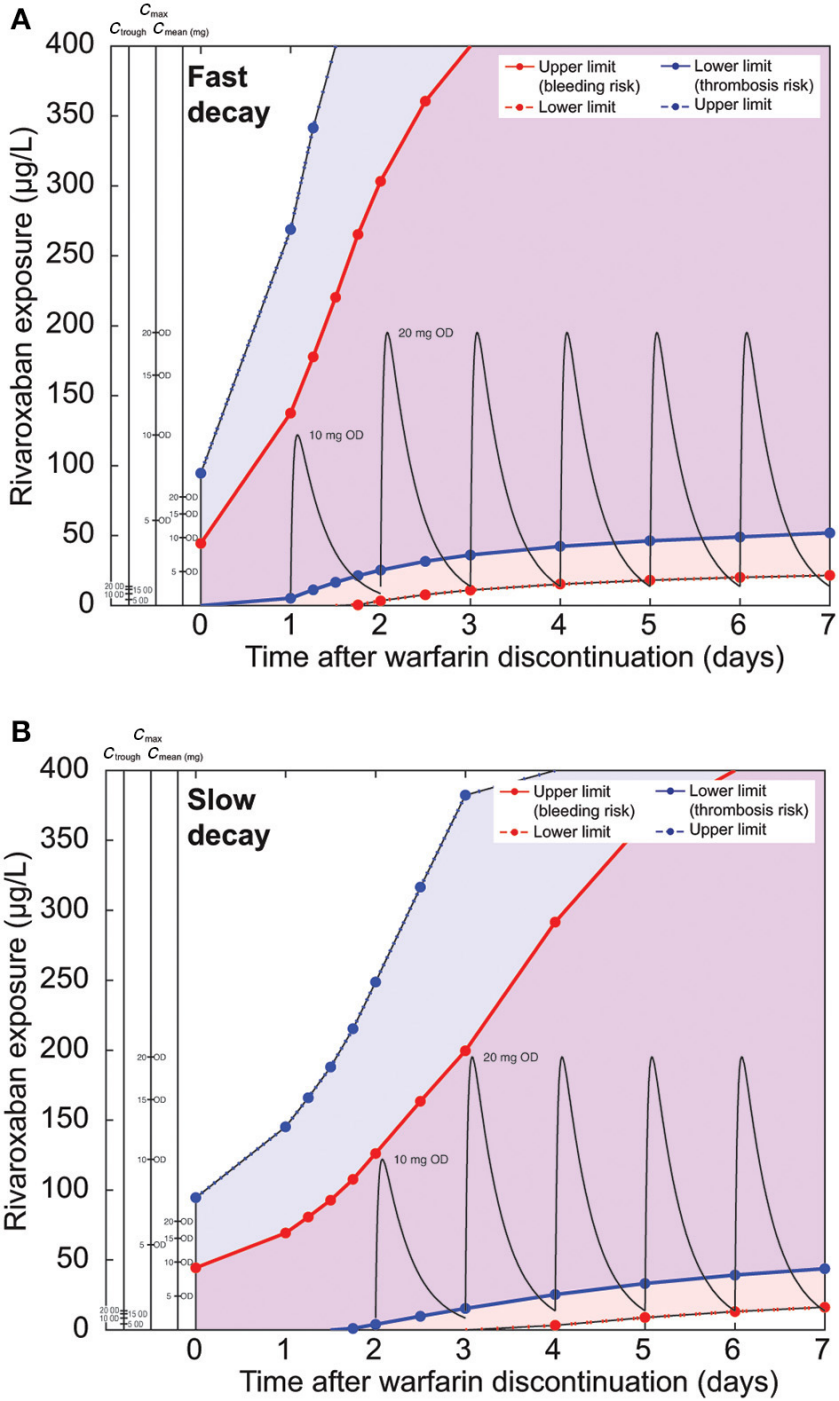
**Comparison of nomograms for patients with a starting INR of 2.5, assuming (A) fast or (B) slow warfarin decay**. Pharmacokinetic curves for rivaroxaban 10 and 20 mg OD are drawn in black. (Note: please refer to legend of Figure 6 for further details.)

We then investigated the impact of the PK half-life of warfarin. We repeated the simulations for Caucasian patients starting at an INR of 2.5 but used half-life combinations with the slowest and fastest decay rather than the mean values used previously (Figures 6–8; mean half-lives: S-warfarin 29 h, R-warfarin 20 h). For fast decay, we used the following half-lives: S-warfarin 18 h and R-warfarin 45 h (Figure 9A). For slow decay we used half-lives for S-warfarin of 52 h and for R-warfarin of 70 h (Figure 9B). As expected, a faster PK decay of warfarin broadened the safety and efficacy corridor early (Figure 9A); the dosing schedule as suggested for normal warfarin half-lives (Figure 6) complies with the safety and efficacy corridor in this scenario. The slower decay led to a slower broadening of the corridor this time, suggesting a delayed start of rivaroxaban dosing on day 2 (Figure 9B).

Finally, we repeated the calculations for the Caucasian base case starting at an INR of 2.5 with *in silico* patients characterized by the over- or under-expression of various coagulation factors (Table 3; Supplementary Material 2). Only non-vitamin K-dependent factors (Factors I [fibrinogen], V, VIII, and XI) were chosen for this analysis because these can be considered to be independent of warfarin. Variations in Factors VIII or XI showed little impact on the overall appearance of the safety and efficacy corridors even at extreme over- or under-expression (Supplementary Material 2). At the lower end of the Factor I and V concentrations, the corridors were shifted toward lower levels, illustrating an overall increase in bleeding risk. At the upper extreme of Factor V over-expression the lower efficacy threshold was clearly increased. Factor I increases had little impact.

## Discussion

We used a computational coagulation model to investigate changes in the combined coagulation effect during the switch from warfarin to rivaroxaban in Caucasian and Japanese *in silico* patients. Our model was an extended version of a previously evaluated ODE-based coagulation model (Burghaus et al., 2011). We added a vitamin K-dependent factor turn-over model and an effective warfarin action and decay module. The model was structurally similar to published models by Wajima et al. (2009) and Orfeo et al. (2010, 2011). Our model also represents warfarin enantiomers and enantiomer-specific differences in warfarin clearance between Caucasian and Japanese patients (Takahashi et al., 2006). Warfarin PK is modeled via effective warfarin concentrations with respect to the resulting inhibition of the synthesis of vitamin K-dependent factors. Warfarin clearance is implemented as a decay of the inhibition of synthesis.

Our model only implicitly takes into account that the dose–response relationship of warfarin is dependent on genetic and environmental factors such as food and concomitant drugs. These factors may influence the PK (e.g., the decline of concentrations because of metabolic clearance of both S-warfarin and R-warfarin enantiomers) and the PD of warfarin (e.g., by provision of higher concentrations of vitamin K, which counteracts VKA action; Ansell et al., 2008).

To evaluate the warfarin action and decay module, we compared the simulated decay of warfarin effect on INR with clinical data from a warfarin discontinuation study (Figure 3) (White et al., 1995) and obtained a high level of agreement. Because the model parameters were not fitted to match the clinical discontinuation data used by White et al. (1995) but taken from independent published experimental data (Wittkowsky, 2003), the excellent agreement between clinical data and the model can be considered a validation of the model extension.

When we simulated the effect of rivaroxaban exposure combined with the residual warfarin effect after discontinuation of warfarin, we demonstrated a strong synergistic effect. This finding can be explained by the fact that inhibition by warfarin leads to lower levels of Factor X, which resulted directly in lower levels of free Factor Xa and Factor Xa–Factor Va (prothrombinase complex). This inhibition is further enhanced by the fact that both its substrate thrombin and the Factor X activation systems (intrinsic and extrinsic tenase) are present at lower levels. Consequently, there is a higher level of inhibition when both free Factor Xa and Factor Xa–Factor Va are targeted by rivaroxaban (Perzborn et al., 2005). As expected, the synergistic effect of residual warfarin action on the rivaroxaban effect decreased over time and became negligible for a typical *in silico* patient after 2–3 days if the treatment with warfarin was discontinued at an INR of 2.5, in-line with guidelines for the treatment of patients with atrial fibrillation treated for the prevention of stroke (Ageno et al., 2012). For simulations with a long warfarin half-life the synergistic effect lasted longer.

To relate our simulations to clinically relevant scenarios for the switching of a patient from warfarin to rivaroxaban, we applied a set of efficacy and safety criteria. These criteria were established previously to derive the therapeutic window for rivaroxaban. The criteria were based on a comparison with warfarin effects under several simulated coagulation scenarios (Table 3) and allowed the identification of rivaroxaban exposure and related doses applied during the decay phase of the warfarin effect complying with the intended therapeutic effect. The outcome of the analysis was translated into nomograms for typical Caucasian and Japanese patients (Figures 6, 7), higher and lower initial INR starting values (Figure 8), and patients with high and low warfarin half-life (Figure 9). These scenarios were chosen to show robustness of the results within the range of warfarin-related variability and slightly exceeding its therapeutic range. Examples for rivaroxaban dosing schedules that fulfill the therapeutic criteria are indicated within the nomograms. Of note, although not depicted explicitly, these graphs also allow evaluation of the potential impact of pharmacokinetic variability of rivaroxaban. Depending on the simulated conditions, our analysis suggested different bridging schedules. Extreme situations such as starting at a sub-therapeutic INR value of 1.5 allowed a flat dosing of the target rivaroxaban dose for Caucasian patients (rivaroxaban 20 mg once daily) immediately after discontinuation (Figure 8A). Simulations for typical Caucasian patients starting at an INR of 2.5 suggested starting rivaroxaban treatment with a bridging dose of 10 mg on day 1 after discontinuation of warfarin followed by standard treatment with 20 mg once daily from day 2 onwards. For patients starting at a higher INR value of 3.5 (Figure 8B) as well as patients starting at INR 2.5 but with a long warfarin half-life (Figure 9B), the suggested rivaroxaban schedule was shifted by 1 day.

Compared with Caucasian patients, Japanese patients have a higher exposure to rivaroxaban owing to their lower clearance via cytochrome P450 2C9 (Hori et al., 2012). Population differences in warfarin-relevant enzymes, such as vitamin K epoxide reductase complex subunit 1 (VKORC1) and gamma-glutamyl carboxylase (GGCX) (Kimura et al., 2007), were not explicitly included in the model but are implicitly represented by the adjustment of the initial steady-state condition in the Japanese version of the coagulation model. For typical Japanese patients (Figure 7), the suggested bridging dose on days 1 and 2 was rivaroxaban 10 mg once daily followed by 15 mg once daily from day 3 onwards. The lower final doses of rivaroxaban match the Japanese label (Bayer Yakuhin Ltd., 2012; Hori et al., 2012). The rationale to test the reduced dose of rivaroxaban specifically in Japanese patients was based on the higher exposure data in this population and the lower warfarin target INR in Japanese clinical practice (Hori et al., 2012). It should also be noted that the reduced dose of 15 mg once-daily rivaroxaban is only approved in Japan but this dosing regimen may not be applicable in other Asian countries. All bridging scenarios described above (reduced bridging dose followed by standard treatment) comply with the stricter criteria for patients at high risk of bleeding. For all patients without a high risk of bleeding, flat dosing schedules starting with the target rivaroxaban dose instead of the reduced bridging dose comply with the safety and efficacy criteria.

We also simulated the effect of factor deficiencies in non-vitamin K-dependent factors (Factors I, V, VIII, and XI), because these would not necessarily be compensated for by titration of warfarin to a target INR. The predicted safety and efficacy corridors changed only for severe over- or under-expression of these factors (Supplementary Material 2). We conclude that this finding is only relevant for patients who previously showed clinical symptoms. This monovariate sensitivity analysis also indicated that our findings are robust against plausible variability of factor concentrations in non-hemophilic and non-thrombophilic patient populations.

We restricted our assessment to the typical simulation behavior of individual patients, representing the mean plasma PK after rivaroxaban dosing as well as typical short, intermediate, and long half-lives of warfarin. As a consequence, our simulations do not represent variability in a population of patients switching from warfarin to rivaroxaban. In clinical practice, clinicians will encounter higher as well as lower INR values in their patients. We complemented our analysis of typical behavior with an investigation of the potential impact of different (baseline) expression levels of coagulation factors not dependent on vitamin K. During model development, model parameters were selected such that they are aligned with respective (microscopic) parameters provided by the literature and that simulated systemic properties (e.g., coagulation times, amount of thrombin generated, response to different types of pharmacologic interventions) are aligned with these systemic properties reported in the literature.

Our model-based assessment provides mechanistic insight into the interaction of warfarin and rivaroxaban during the switching period between the two treatments. It supports product information for rivaroxaban in the United States and Europe (US Prescribing Information; European Summary of Product Characteristics for rivaroxaban) (Bayer Pharma, 2014; Janssen Pharmaceuticals Inc., 2014). When switching patients with atrial fibrillation from warfarin to rivaroxaban, the US Prescribing Information recommends to discontinue warfarin and if the INR is below 3.0 to start rivaroxaban on the following day (Janssen Pharmaceuticals Inc., 2014). The European Summary of Product Characteristics broadly concurs with this advice (the recommended INR is ≤3.0). For patients treated for deep vein thrombosis and/or pulmonary embolism and for prevention of recurrent deep vein thrombosis and pulmonary embolism, the recommendation is to discontinue VKA therapy and initiate rivaroxaban when the INR is ≤2.5 (Bayer Pharma, 2014). These INR-based switching strategies provide a clinically feasible approach to maximize safety and efficacy during the switching period by characterization of patients prior to switching by mean of the well-established INR value. Our simulations for initial INR values of 2.5 versus 3.5 illustrate the relevance of the INR threshold (Figures 6, 8B). In the latter case, simulations suggested initiation of rivaroxaban therapy later than day 1 after discontinuation, which would be the practical outcome of an INR value of 3.5 in the clinic. As discussed above, efficacious and safe therapies with different anticoagulants usually require different levels of biomarkers, or even different biomarkers to characterize the drug effect (for example, aPTT is a more relevant test than PT for direct thrombin inhibitors). INR is appropriate only for measuring the anticoagulant activity of VKAs. It is not appropriate for assessing anticoagulation with rivaroxaban (Bayer Pharma, 2014; Janssen Pharmaceuticals Inc., 2014) or coagulation status during the transition from warfarin to rivaroxaban (Kubitza et al., 2005a,b). The use of INR only prior to dosing of rivaroxaban consistently implements this fact.

### Learning points

The efficacy and safety nomograms defined by our simulations support the switching strategies provided in the Summary of Product Characteristics and Prescribing Information for rivaroxaban (Bayer Pharma, 2014; Janssen Pharmaceuticals Inc., 2014). There is no biomarker that predicts clinical consequences of the combined effect of rivaroxaban and warfarin in the transition phase. Large-scale clinical trials focusing on clinical outcomes of different bridging options at low or high INR in the therapeutic range of warfarin are not feasible. Simulations of different clinically realistic scenarios are presented and provide clinicians with a mechanistic pharmacologic rationale for these strategies and underline the relevance of the recommendations.

### Conflict of interest statement

Rolf Burghaus, Anke Sensse, Wolfgang Mueck, Takahiko Tanigawa, and Jörg Lippert are employed by Bayer Pharma AG. Katrin Coboeken, Thomas Gaub, Christoph Niederalt, Hans-Ulrich Siegmund, and Wolfgang Weiss are employed by Bayer Technology Services GmbH.

## Supplementary material

Supplementary Material 1 **Information pack including specification of model parameters and codes used for the reproduction of study simulations**. Simulations were generated using the following versions of the BTS software platform: MoBi® 2.3, MoBi® Toolbox for Matlab® 2.3. This pack provides information on how to download the software, how to re-run the simulations and how to reproduce the graphical representations.

Supplementary Material 2 **Nomograms for *in silico* patients with different factor abnormalities, including over-expression and under-expression of the non-vitamin K-dependent factors I, V, VIII, and XI**. Factor deficiencies are indicated by x-fold change compared with normal values and range from 0.01 to 5. The x-axis describes the time after discontinuation of warfarin and the y-axis corresponds to rivaroxaban exposure. Blue and red lines correspond to the edges of the safety and efficacy region. The strong extrinsic trigger (TF = 10^−11^ M) was assumed to be the relevant safety scenario. The weak extrinsic trigger (TF = 10^−14^ M) was considered to be the relevant efficacy scenario. Rivaroxaban exposure above the dashed red line is considered to be safe and exposure below the dashed blue line is considered to be efficacious for most patients. The region between the solid red and solid blue lines describes the exposure range to which patients with a high risk of bleeding or thrombosis should be dosed. Patients with a high risk of bleeding should stay below the exposure limit represented by the solid red line. Patients with a high risk of thrombosis should stay above the exposure limit represented by the solid blue line. Alternative y-axes convert rivaroxaban exposure into mean plasma concentration (*C*_mean_), maximum plasma concentration (*C*_max_), and minimum plasma concentration (*C*_trough_) values for the different dosing regimens studied (5, 10, 15, and 20 mg once daily [OD]). These nomograms do not account for pharmacokinetic variability or uncertainty in parameters. However, the potential impact of pharmacokinetic variability of rivaroxaban can also be evaluated from these graphs although this is not depicted explicitly. To extract text information from the figure panels, such as labels of x-and y-axes or other parameters, please zoom into the panel of interest. INR, international normalized ratio.
