# Supplementary material for: Computational investigation of potential dosing schedules for a switch of medication from warfarin to rivaroxaban—an oral, direct Factor Xa inhibitor
Source: Front Physiol. 2014 Nov 7;5:417. doi: 10.3389/fphys.2014.00417 (PMC4224077; doi:10.3389/fphys.2014.00417)
Supplement: Supplementary Material 1 — Information pack including specification of model parameters and codes used for the reproduction of study simulations. Simulations were generated using the following versions of the BTS software platform: MoBi® 2.3, MoBi® Toolbox for Matlab® 2.3. This pack provides information on how to download the software, how to re-run the simulations and how to reproduce the graphical representations. [file SupplementaryMaterial1.ZIP › How to handle the submitted electronic information.doc]

# How to handle the submitted electronic information

For the Publication “Computational investigation of potential dosing schedules for a switch of medication from warfarin to rivaroxaban – an oral, direct factor Xa inhibiton”, the Bayer Technology Services (BTS) software tools MoBi^®^ and the MoBi^®^ Toolbox for Matlab^®^ were used.

# 1 Content of the Switch of medication package

To access the submitted information, please unzip the Switch of medication package “Switch of medication.zip”. This will create the files and the folders described in the following.

- “How to handle the submitted electronic information.doc”: Word file of this document,
- Wrapper.m List of all matlab calls necessary to reproduce the result and figures
- Input.xls Excel file, which defines the therapies and patients which are simulated
- Folder BCM_Code: Contains the relevant code used to generate the results and the figures
- Folder Data: Contains the relevant data used for the figures

# 2 SoftwarePackage

Simulations were generated using the following versions of the BTS software platform: PK-Sim^®^ 4.2, MoBi^®^ 2.3, MoBi^®^ Toolbox for Matlab^®^ 2.3. To download the software, visit [www.systems-biology.com](http://www.systems-biology.com/). For license issues, please contact info@systems-biology.com. Matlab^®^ including the Matlab^®^ Statistics Toolbox (versions R2007 to R2012b were tested in-house for compatibility, http://www.mathworks.com/products/matlab/).

# 3 How to reproduce the results reported

The following description provides details on how to re-run the simulations and how to reproduce the graphical representations. In order to re-run simulations or plots, please make sure that the required software is installed, see Section 2

Unzip the Switch of medication package and store it locally on a computer drive. The content is described in detail in Section 1.

Hereafter, the path of the unzipped package is called <PackagePath> (please include a backslash “\” at the end).

The simulation functionality of the MoBi^®^ Toolbox for Matlab^®^ (called Matlab^®^ code hereafter) is called within Matlab^®^ by commands listed below

Select the <packagepath> as current folder.

## 3.1 Parameter settings and Path

Add the Matlab^®^ code of the installed MoBi Matlab Toolbox with all subdirectories to the Matlab^®^ path:

addpath(genpath('C:\Program Files\BTS Products\MoBi Toolbox for Matlab 2_3'));

Add the Matlab^®^ code of the Switch package

version_path=[cd filesep 'BCM_Code' filesep];

addpath(version_path);

Define the Parameter which contains the model pathes:

sxml.PPP=[version_path 'PPPinVitro_3_Files' filesep 'PPPinVitro_3.sxml'];

sxml.PPP_Reservoir=[version_path 'PPPinVitro_3_Reservoir_Files' filesep 'PPPinVitro_3_Reservoir.sxml'];

sxml.PRP=[version_path 'PRPinVitro_3_Files' filesep 'PRPinVitro_3.sxml'];

sxml.Warfarin=[version_path 'WarfarinPlusC_files\WarfarinPlusC.sxml' ];

## 3.2 Reproduce simulation results

% result for figure 2

prepareWarfarinDecay(sxml.Warfarin,sxml.PPP);

% result for figure 5C

prepareWarfarinDecayJapanesRatio(sxml.Warfarin,sxml.PPP);

% result for figure 4, 5A (Caucasians)

runWarfarinSwitch(sxml,1,2,[1 2 4 6 7]);

% result for figure 5B (Japanese)

runWarfarinSwitch(sxml,1,1,[1 4 6 7]);

% result for supplementary,

runWarfarinSwitch(sxml,1,3:52,[1 4 6 7]);

## 3.3 Reproduce figures

```
close all;
if ~exist('figures','dir')
    mkdir('figures')
end

% Input parameter DosisRange m.file
tau_fast=1;
tau_typical=2;
tau_slow=3;
INR_1p5=1;
INR_2p5=3;
INR_3p5=5;
iP_cauc=2;
iP_jap=1;


% Fig2
plotWarfarinDecay;
set(gcf,'position',[208   150   984   793]);
print('-depsc', ['figures/Warfarin_SwitchFig2.eps']);

% Fig3
plotWarfarin(sxml)
print('-depsc', ['figures/Warfarin_SwitchFig3.eps']);

% Fig 4
plot_synergisticEffect
print('-depsc', ['figures/Warfarin_SwitchFig4.eps']);

% Fig 5 Caucasian
plot_Switch_Slope(2,3,2)
print('-depsc', ['figures/Warfarin_SwitchFig5_A.eps']);

% Fig 5 Japanese
plot_Switch_Slope(2,3,3)
print('-depsc', ['figures/Warfarin_SwitchFig5_B.eps']);

% Fig 5 C Ratio
plotWarfarinDecayJapanesRatio
print('-depsc', ['figures/Warfarin_SwitchFig5_C.eps']);


% Fig 6
plot_Switch_Dosis_Range(tau_typical,INR_2p5,iP_cauc,false)
print('-depsc', ['figures/Warfarin_SwitchFig6.eps']);

% Fig 7
plot_Switch_Dosis_Range(tau_typical,INR_2p5,iP_jap,false)
print('-depsc', ['figures/Warfarin_SwitchFig7.eps']);

% Fig 8 A
plot_Switch_Dosis_Range(tau_typical,INR_1p5,iP_cauc,false)
print('-depsc', ['figures/Warfarin_SwitchFig8_A.eps']);

% % Fig 8 B
plot_Switch_Dosis_Range(tau_typical,INR_3p5,iP_cauc,false)
print('-depsc', ['figures/Warfarin_SwitchFig8_B.eps']);

% Fig 9 A
plot_Switch_Dosis_Range(tau_fast,INR_2p5,iP_cauc,false)
print('-depsc', [ 'figures/Warfarin_SwitchFig9_A.eps']);

% Fig 10 B
plot_Switch_Dosis_Range(tau_slow,INR_2p5,iP_cauc,false)
print('-depsc', ['figures/Warfarin_SwitchFig9_B.eps']);
```

## 3.4 Reproduce supplementary figures

```
Input_xls='Input.xls';
Patients=readPatients(Input_xls);
patientRange=4:length(Patients.Names);

for iPatient=patientRange
    try
        figfile=['figures/Warfarin_Switch_' strrep(Patients.Names{iPatient},'.','_')  '.eps'];
        figfile=strrep(figfile,'defficient','deficient');
%         if ~exist(figfile,'file')
            plot_Switch_Dosis_Range(tau_typical,INR_2p5,iPatient,true);
            print('-depsc',figfile);
%         end
    catch exception
        disp(Patients.Names{iPatient});
        disp(exception.message);

    end
end
```
